# Deformation Behavior of Asymmetric Direct Laser Interference Patterning Structures on Hot-Dip Tinned Copper

**DOI:** 10.3390/ma18235278

**Published:** 2025-11-22

**Authors:** Silas Schütz, Sebastian Suarez, Yannik Bautz, Prateek Sharma, Stefan Diebels, Frank Mücklich

**Affiliations:** 1 Department of Materials Science and Engineering, Saarland University, 66123 Saarbrücken, Germanysharmap@hsu-hh.de (P.S.);; 2 Department of Mechanical and Civil Engineering, Helmut Schmidt University, 22043 Hamburg, Germany

**Keywords:** laser structuring, asymmetry, indentation, contact mechanics

## Abstract

Understanding contact mechanics is essential for optimizing electrical and mechanical interfaces, particularly in systems where surface structuring influences performance. This study investigates the mechanical contact behavior of hot-dip tinned copper surfaces modified via Direct Laser Interference Patterning (DLIP). Asymmetric, line-like microstructures with varying periodicities (2–10 µm) and tilt angles (0°, 15°, 30°) were fabricated on both as-received and aged hot-dip tinned copper substrates. The resulting surfaces were characterized using confocal laser scanning microscopy and subjected to indentation testing under controlled loads. Contact mechanical calculations and finite element simulations were employed to determine critical values for plastic deformation onset and to access the real contact area. Results show that structural periodicity, tilt angle, and material condition significantly affect load-bearing capacity and deformation behavior. Notably, intermediate periodicities (e.g., 7.5 µm) on as-received material at 0° tilt exhibited the highest susceptibility to plastic deformation, while aged samples demonstrated improved mechanical stability due to the harder Cu_6_Sn_5_ surface layer, which forms directly after coating and grows during aging until it reaches the surface and no residual tin is left. These findings provide valuable insights into the design of structured contact surfaces for electrical applications, highlighting the importance of tailored surface morphology and material selection.

## 1. Introduction

Contact mechanics plays a pivotal role across a broad spectrum of applications, including biomechanical systems, bearings, machine interfaces, and electrical contacts. Even surfaces that appear nominally flat exhibit inherent roughness, resulting in contact occurring at discrete points across multiple asperities. These localized contact regions often experience elevated pressures and stresses, potentially leading to material yielding or failure. Extensive research has been conducted on single and multi-asperity contact mechanics, as reviewed by Bhushan [1,2] and Ghaednia et al. [3]. Accurate modelling of contact phenomena necessitates detailed knowledge of surface topography and material properties. In electrical applications, contact mechanics modelling is instrumental in predicting electrical behavior, as outlined by Holm [4].

In response to the complexities of contact behavior, surface engineering has emerged as a key strategy for enhancing the reliability and functional performance of electrical interfaces. Through targeted surface modification techniques, electrical contacts can be optimized and stabilized, while frictional properties can be precisely adjusted to either promote secure connection or prevent unintended disconnection. Studies [5,6,7] have demonstrated that deterministic surface topographies outperform stochastic surfaces produced by conventional manufacturing methods. Such engineered surfaces can be fabricated via etching [8], stamping [9] or laser-based techniques. Among laser-based methods, Direct Laser Interference Patterning (DLIP) has emerged as a promising approach. Since its invention, DLIP has evolved rapidly and now enables processing rates up to 1 m^2^/min [10]. DLIP has been shown to reduce both the coefficient of friction [11] and electrical contact resistance [12], making it particularly suitable for contact material optimization. In this study, DLIP-fabricated surfaces with well-defined topography are investigated.

To understand the mechanical behavior of these engineered DLIP surfaces, it is essential to consider their multi-scale roughness characteristics. Microscopically, the surfaces exhibit significant roughness with irregular asperities due to the laser process. On the mesoscale, the line-like features themselves act as elongated asperities. Classical contact mechanics concepts developed for microscale nominally flat surfaces can be extended to these periodic mesoscale structures.

The foundational analysis of elastic contact was conducted by Hertz in 1881 [13], based on assumptions of smooth, nonconforming surfaces, small strains, elastic half-space behavior near the contact region, and frictionless interaction. Greenwood and Williamson later expanded this theory to include plastic deformation, concluding that while individual contact areas remain load-independent, the number of contacts increases with load [14]. Consequently, the real contact area *A_r_* is dependent on the applied load. According to [15], it can be estimated using the relation $A_{r}=F_{N}/H$, where *F_N_* is the normal force and *H* the hardness of the softer material. This expression is particularly relevant for interpreting measurement results and predicting electrical contact resistance, as discussed in [16].

To accurately capture real contact behavior, which often lies between purely elastic and fully plastic regimes, elastic–plastic models are required. Given the complexity of analytical and numerical approaches, finite element analysis is widely adopted for simulating such contacts, as supported by Bhushan [1] and Ghaednia et al. [3].

Considering the interplay between contact area, prediction of resistance and elastic–plastic material response, the present study integrates theoretical modelling with experimental validation to assess the accuracy of contact mechanics predictions and to elucidate observed phenomena in engineered electrical contact surfaces.

## 2. Materials & Methods

### 2.1. Test Specimens

**Figure 1:**
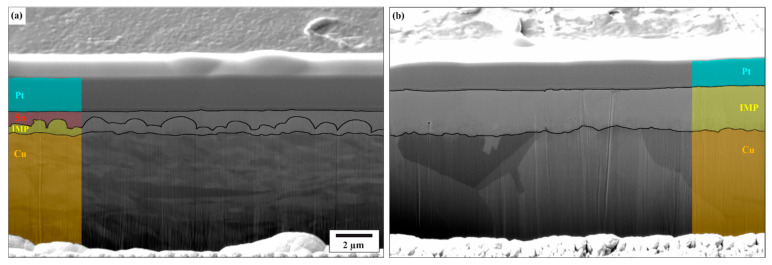
(**a**) As-received and (**b**) aged Sn-coated plates in cross-section. There are indications of the various constituent phases. The intermetallic phases (IMP) consist of Cu_6_Sn_5_ (at the interface to Sn) and Cu_3_Sn (at the interface to Cu). Both cross sections have a Pt layer placed on top to shield the original surface.

Two different types of hot-dip tinned copper platelets were investigated, one in as-received and the other in aged conditions (see Figure 1). For the samples in the as-received condition, the substrate material is CuSn0.15 and for the aged samples it is CuNi3Si1Mg. Immediately following the tinning process, intermetallic phases begin to form at the interfaces: Cu_6_Sn_5_ (η-phase) at the tin interface and Cu_3_Sn (ε-phase) at the copper interface. These phases continued to grow due to aging [17]. In the as-received state, the platelets consisted of the substrate, a tin surface layer measuring 0.52 ± 0.19 µm and an intermediate layer of intermetallic compounds with a thickness of 0.55 ± 0.22 µm. The aging process was accelerated through thermal treatment, resulting in complete consumption of the tin layer. Consequently, the substrate was covered by a 2.19 ± 0.32 µm thick intermetallic layer, with Cu_6_Sn_5_ exposed at the surface and a thin Cu_3_Sn layer at the interface with the copper base.

For sample preparation, tinned copper strips were cut into 20 × 25 mm^2^ platelets and cleaned in an ultrasonic bath with isopropanol for 10 min.

To fabricate inclined microstructures, Direct Laser Interference Patterning (DLIP) was employed as the structuring technique. DLIP operates by splitting a linearly polarized laser beam into two coherent sub-beams, which are then superimposed on the sample surface to generate a periodic intensity distribution. Constructive interference results in intensity maxima, while destructive interference produces minima. The resulting structural periodicity (Λ) is defined by Λ = (λ/2) ∙ sin(θ), where λ is the laser wavelength and θ is the half-angle between the interfering beams. Further details on the method can be found in the literature [18,19].

In this study, an optical head was integrated with an Edgewave InnoSlab PX laser (pulse duration: 12 ps; wavelength: 532 nm) within the RDX 500 Nano picolaser system (Pulsar Photonics, Aachen, Germany). The optical head contains all necessary components to split the primary beam and adjust the focal angles of the sub-beams. Symmetrical line-like structures (α = 0°) were fabricated using periodicities of 2 µm, 5 µm, 7.5 µm and 10 µm. To introduce asymmetry, the sample was tilted by a defined angle α (15° or 30°), as reported in [20,21], resulting in sawtooth-like structures. With these distinct tilt and the previously specified periodicities, asymmetric sawtooth-shaped structures were produced. Across all configurations, the structural depth was maintained at 1.5 µm within a defined tolerance. In addition to the patterned samples, unmodified reference specimens were also analyzed. Prior to further characterization, all samples were cleaned in an ultrasonic bath with isopropanol for 10 min.

### 2.2. Surface Characterization

The reference samples were examined using focused ion beam microscopy (FIB) in conjunction with scanning electron microscopy (SEM, Thermo Fisher Helios G4 PFIB CXe, Waltham, MA, USA) The pictures were acquired with a secondary electron detector with 5 and 10 keV.

The DLIP-fabricated structures, along with reference samples, were characterized using confocal laser scanning microscopy (CLSM, Olympus OLS 4100, Tokyo, Japan). Imaging was performed with a 405 nm laser and a 50× objective lens (N.A. = 0.95), yielding a vertical (*Z*-axis) resolution of 10 nm and a lateral (X-Y) resolution of 120 nm. Multiple scans were acquired for each sample, and representative 3D reconstructions were exported.

Each image was further evaluated using three line profiles taken from the top, middle, and bottom regions. In accordance with ISO 21920, nesting indices Nic and Nis were applied, and set to zero and one-quarter of the periodicity, respectively, to separate waviness from roughness. In this context, the laser-induced surface profile is interpreted as waviness, while micro-roughness is of less relevance here. From the filtered profiles, two key parameters were extracted: skewness (Rsk) and kurtosis (Rku). They describe the shape of the surface profile and are calculated using the third and fourth powers of the root mean square deviation, respectively. Skewness quantifies the asymmetry of the amplitude distribution (Rsk = 0 corresponds to a symmetric distribution), whereas kurtosis reflects the sharpness of profile peaks by measuring the steepness of the amplitude density curve (Rku = 3 corresponds to a Gaussian normal distribution). Additionally, the core roughness (Rk), referring to the laser-induced surface features, was determined. This parameter is closely associated with the material’s load-bearing capacity and wear resistance, where higher Rk values indicate reduced load-bearing performance and increased susceptibility to wear, due to a larger amount of surface asperities.

### 2.3. Indentation Tests

Indentation experiments were conducted using a custom-built test rig, comprising a sample holder mounted on a linear motion stage and a force sensor equipped with a counterbody fixture. A detailed description of the setup is provided in [22]. The primary focus of this study was on the mechanical interactions during indentation. 100Cr6 steel balls with a diameter of 6 mm and a root mean square surface roughness (Sq) of 0.15 ± 0.02 µm were used as counterbodies. Indentation tests were carried out under varying normal loads of 1, 3, 5, and 10 N. Each test condition was repeated three times to ensure reproducibility. Some electrical measurements were performed only to supplement the mechanical results. These measurements employed the four-wire method under dry-circuit conditions (0.1 A) at a normal load of 3 N.

Following indentation testing, the contact zones on the samples were analyzed using confocal laser scanning microscopy (CLSM). As illustrated in Figure S1 in the Supplementary Information, intensity images were utilized for the geometric assessment of indents on structured samples. For reference samples, height images were employed due to insufficient contrast in the intensity images.

### 2.4. Contact Mechanical Calculations

To gain deeper insight into the contact mechanics, additional calculations were performed. As an initial step, the force per topographical maximum was estimated using the relation

(1) $$P_{N}^{\left(max\right)}=\frac{P_{N}}{n^{\left(max\right)}}$$

where $n^{\left(max\right)}$ is the number of topographical maxima within the contact zone, determined from experimental data.

**Figure 2:**
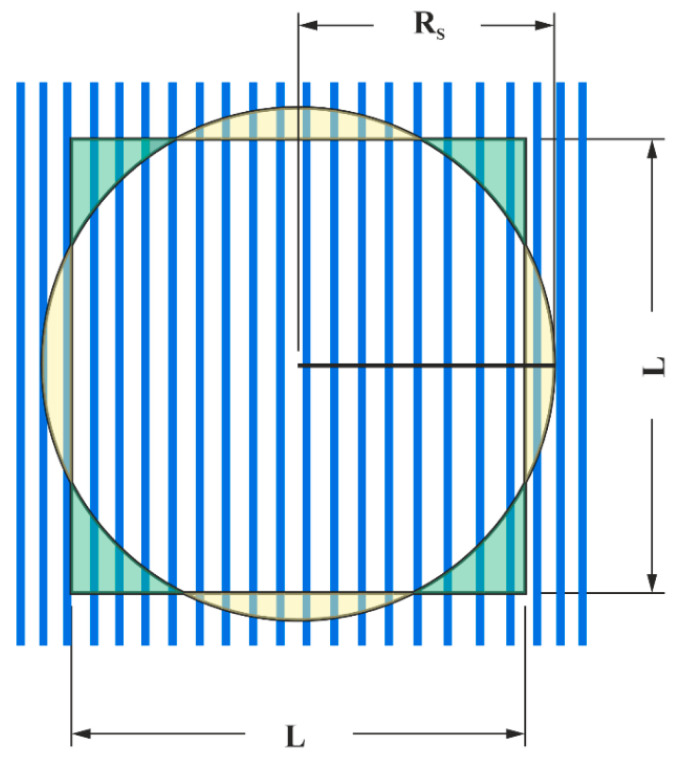
Schematic representation of the geometric approach used to relate the spherical contact area to the rectangular assumptions found in the literature.

Subsequently, the critical load per maximum required to initiate plastic deformation was calculated for the as-received material. As previously discussed, the line-like surface structures can be interpreted as elongated asperities at the mesoscale, while the counter-body can be approximated as a flat surface due to its comparatively larger diameter. This configuration allows the contact at a single topographical maximum to be modelled as a cylindrical Hertzian contact. According to Ghaednia et al. [3], the critical load for plasticity in such a contact is given by:

(2) $$P_{C}=\frac{\pi *R{*\left(C*S_{y}\right)}^{2}}{E}*L$$

where *R* is the radius of curvature of the surface asperities, *S_y_* is the yield strength, *E* is the elastic modulus, and *L* is the contact length. The constant *C* depends on the Poisson’s ratio *ν* of the material and is defined as $C\;=\;1.164\;+\;2.975*\nu \;-\;2.906*\nu^{2}$ [3]. In this study, the counter-body is spherical, resulting in a circular contact zone. To reconcile this with the rectangular contact assumption in Equation (2), the contact length *L* is replaced by $\sqrt{\pi }*R_{S}$, where *R_S_* is the apparent contact radius. This equivalence is based on geometrical considerations (see Figure 2) where the area of a square and a circle are matched.

Assuming $R_{s}=\;1$ and $L=\sqrt{\pi }$, the area of both shapes is equal, thus

(3) $$L=\sqrt{\pi }*R_{s}$$

for any contact radius *R_S_*. Substituting into Equation (2) yields:

(4) $$P_{C}=\frac{\pi *R{*\left(C*S_{y}\right)}^{2}}{E}*\sqrt{\pi }*R_{s}$$

Further, the critical real contact area for the onset of plasticity for the as-received material and 3 N contact force was calculated as

(5) $$A_{t}=\frac{P_{N}}{P_{C}}*2b_{C}*\sqrt{\pi }*R_{s}$$

where *P_N_* is the applied load and *b_C_* is the critical contact radius, given by

(6) $$b_{C}=\frac{2RCS_{y}}{E}$$

according to Ghaednia [3].

All calculations assume a homogeneous Sn surface layer, neglecting the substrate. The material parameters used are summarized in Table 1.

Additional parameters, such as the radius of curvature of the surface structures *R* and contact radius *R_S_*, were obtained from CLSM analysis. For *R_S_*, the smaller diameter of the elliptical indent (see Figure S1 in the Supplementary Information) was used. This choice is justified by two considerations: (1) the elliptical shape likely results from unintended sliding due to misalignment, making the smaller diameter a more accurate representation of the initial contact area; and (2) underestimating the contact radius leads to conservative (i.e., higher) estimates of the critical load for plastic deformation.

### 2.5. Simulations

To further investigate the contact mechanics and support the interpretation of the experimental findings, finite element simulations were performed using Abaqus (Dassault Systèmes, Vélizy-Villacoublay, France, 2023). Surface structures were reconstructed based on profilometric data, incorporating measured structure depths and the slopes adjacent to topographical maxima as input parameters. Simulations were conducted for periodicities of 5 µm, 7.5 µm, and 10 µm at a tilt angle of 0°, and additionally for tilt angles of 15° and 30° for the 5 µm periodicity.

In the absence of reliable experimental data for the coating material, a bilinear elastoplastic material model was adopted. The elastic modulus was taken from literature, while the yield stress and hardening modulus were adjusted empirically to achieve a realistic indentation depth under a constant contact force of 3 N, using the 5 µm/0° configuration. The aim of this calibration was to obtain a physically plausible material response rather than a unique parameter set. Although the resulting parameters are not uniquely defined, they lie within a reasonable range reported for similar materials, ensuring that the simulated deformation mechanisms remain representative of the expected behavior. Two distinct parameter sets were established to approximate the behavior of the as-received and aged materials.

Due to the empirical nature of the material model calibration, the absolute simulation results are not directly comparable to experimental values. Additionally, frictional effects were not incorporated into the simulations. Prior studies [25,26] have shown that friction becomes increasingly significant for indentation depths where substrate effects cannot be neglected. Although these investigations focused on polymers and nanoindentation, the findings may be applicable to the present study, given the disparity in hardness between the coating and the substrate. However, qualitative comparisons between simulation configurations are valid, and the observed trends can be meaningfully correlated with experimental data. Post-unloading geometric analysis of the simulated indents was performed to isolate plastic deformation, consistent with the experimental methodology. In addition to the circular indent diameter (as assumed in the idealized model), the real contact area was also evaluated. To achieve this, the spatial distribution of contact forces was exported during load application and visualized using a 2D heatmap. The real contact area was then identified through image analysis, defined as the region exhibiting a contact force greater than zero.

## 3. Results & Discussion

### 3.1. Surface Characterization

**Figure 3:**
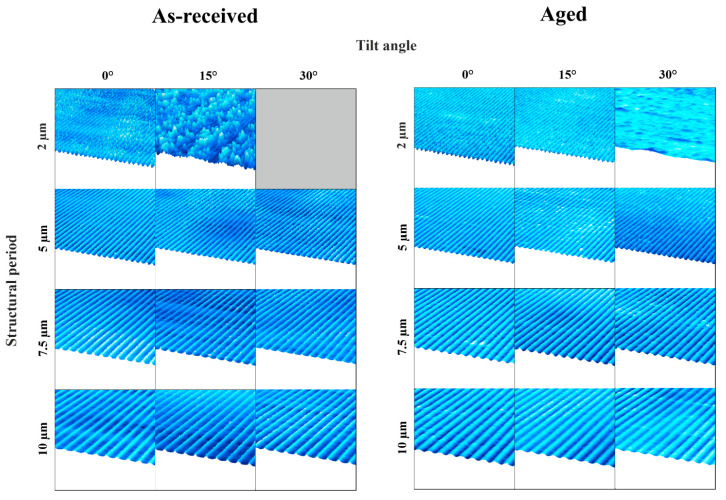
Confocal laser scanning maps of all the structured samples.

Figure 3 presents the 3D reconstructions obtained from CLSM data. In contrast to conventional DLIP structures fabricated at normal incidence (0°), the slopes adjacent to the topographical maxima exhibit a distinct inclination. These images also highlight limitations in achieving certain parameter combinations. Specifically, the fabrication of small periodic structures on tilted substrates proved to be particularly challenging. As the tilt angle increases, reflection effects become more pronounced due to the angle-dependent nature of reflectivity. This dependency varies with the polarization state of the incident laser light [27]. In this study, p-polarized light (i.e., polarized parallel to the plane of incidence) was employed, which exhibits a more significant angular dependence in reflectivity within the relevant range. In DLIP, laser beams inherently strike the surface at an angle, and variations in reflection and absorption are typically compensated by adjusting the overall laser power. However, for tilted samples, the two interfering beams impinge at different angles, resulting in unequal absorption. This discrepancy cannot be corrected by a uniform power adjustment; instead, individual beam modulation would be required, which was not feasible with the current setup. Consequently, the disparity in absorption increases with tilt angle, hindering the formation of well-defined structures with small periodicities.

Despite these challenges, successful fabrication of 2 µm periodic structures on aged material tilted at 15° was achieved. This success is attributed to the distinct surface properties of the aged material, particularly the presence of Sn and Cu_6_Sn_5_ phases. Notably, Cu_6_Sn_5_ has a melting point nearly twice that of Sn (Cu_6_Sn_5_: 415 °C [28], Sn: 232 °C [29]), which enhances the material’s response to laser structuring. Additionally, the aged material exhibits a slightly higher surface roughness (Sq(aged) = 0.21 ± 0.04 µm) compared to the as-received material (Sq(as-received) = 0.20 ± 0.07 µm promoting better laser energy absorption and facilitating the structuring [30]. An influence of the substrate can be precluded, since the structures do not exceed the coating thickness.

To ensure structural homogeneity, tilt angles that result in irregular or poorly defined features are not further investigated. Additionally, tilt angles exceeding those previously specified were not considered. As a result, the 2 µm periodic structure at 30° tilt on the as-received material was not fabricated. Additionally, the 2 µm/15° structure on the as-received material and the 2 µm/30° structure on the aged material are excluded from further analysis due to insufficient pattern quality.

**Figure 4:**
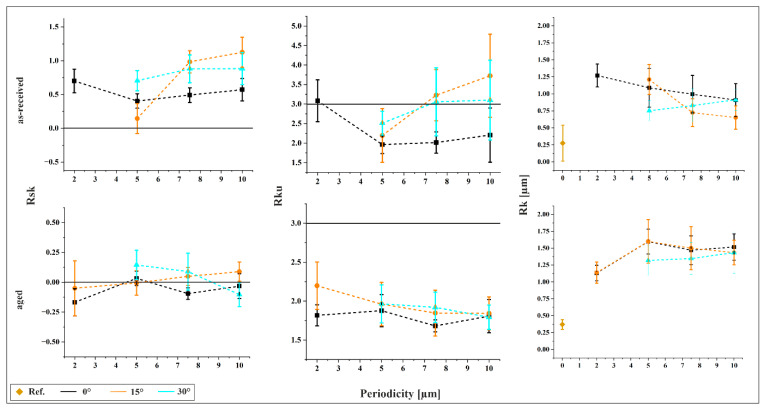
Surface parameters skewness (Rsk), kurtosis (Rku), and core roughness (Rk) for all structured samples.

Figure 4 presents the surface parameters skewness (Rsk), kurtosis (Rku), and core roughness (Rk) for all structured samples. For comparison, Rk is also shown for the reference sample. The as-received material exhibits a positive skewness, indicating a surface dominated by peaks and a relatively low load-bearing capacity. This reduced capacity is further supported by the Rk values, which are consistently higher than that of the reference. A decreasing trend in Rk is observed with increasing inclination, suggesting improved load-bearing capacity. The lowest Rk is found for the 10 µm/15° structure. In the aged material, Rk remains higher than the reference across all structures, with the minimum value observed for 2 µm/15°. Unlike the as-received condition, Rk tends to increase with feature size, indicating a decline in load-bearing performance. Skewness values for aged samples are narrowly distributed around zero, implying a balanced topography with comparable proportions of peaks and valleys.

Kurtosis values below 3 across all structured samples suggest that surface features are not sharply pronounced, resembling a zigzag-like profile. A decreasing trend in Rku with increasing feature size indicates a more irregular surface morphology. Additionally, lower tilt angles tend to produce lower kurtosis values. For the as-received material, Rku increases with periodicity, exceeding 3 in several cases. Notably, the 10 µm/15° structure reaches a Rku of 3.7, indicative of a surface profile sharper than a sine wave.

### 3.2. Geometric Analysis of the Indents

**Figure 5:**
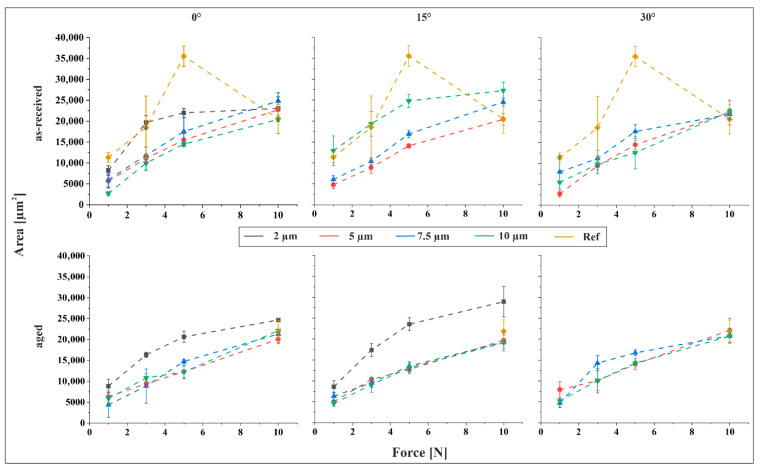
Indent size as a function of force for the as-received and aged material, for 0°, 15° and 30° tilt angle.

**Figure 6:**
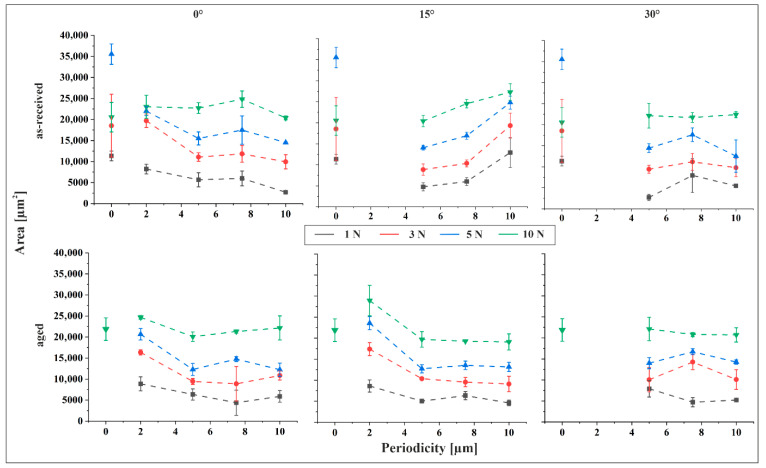
Indent size as a function of periodicity for the as-received and the aged material, for 0°, 15° and 30° tilt angle.

The indent areas derived from geometrical analysis are depicted in Figure 5 as a function of applied force and in Figure 6 as a function of periodicity, with the tilt angle held constant in each plot. For the aged material reference, the contact zone was discernible in CLSM images only at a normal force of 10 N. This can be attributed to the significantly lower core roughness of the reference sample, approximately one-third that of the structured specimens, resulting in a substantially higher load-bearing capacity. Consequently, no visible indents were observed at lower forces. In contrast, for the as-received material reference, the contact zone was evident even at lower forces. This is explained by the markedly lower hardness of Sn compared to Cu_6_Sn_5_ (Sn: 0.22 GPa vs. Cu_6_Sn_5_: 6.38 GPa [23]), with Sn being nearly 30 times softer.

Figure 6 illustrates the influence of structural periodicity on indent size. A comparison of the slopes Figure 5 and Figure 6 reveals that normal force exerts a more pronounced influence on indent size than structural periodicity. As expected, indent size increases consistently with rising force across all measurement series. An exception is observed in the reference sample of the as-received material, where the indent size at 5 N exceeds that at higher forces, including 10 N. This anomaly suggests a potential measurement error. Supporting this interpretation, the trend of increasing indent size with force resumes when the 5 N data point is excluded, and the remaining values fall within the expected range for the as-received reference. Consequently, the 5 N value is treated as an outlier.

In general, indent size decreases with increasing feature size across most parameter combinations. However, a distinct deviation is observed in the 15° tilted as-received material, where indent size increases with feature size at all applied forces, frequently surpassing the reference values. This behavior can be interpreted through the surface characterization data. The 15° tilted as-received samples exhibit the most pronounced increase in both skewness and kurtosis with increasing periodicity. Elevated skewness indicates a surface dominated by peaks, while higher kurtosis reflects sharper surface features. Both characteristics are associated with reduced load-bearing capacity, thereby facilitating deeper indentation. Consequently, the observed increase in indent size with feature size in this specific configuration is consistent with the surface morphology. For the aged material, the largest indents are consistently observed at a periodicity of 2 µm, particularly in the 2 µm/15° structured samples under a 10 N normal force. In some cases, these indents surpass those of the reference sample. Apart from this, the indent size measured on the aged reference at 10 N, the only load where contact was detectable, is comparable to that observed on the structured samples under the same normal force.

**Figure 7:**
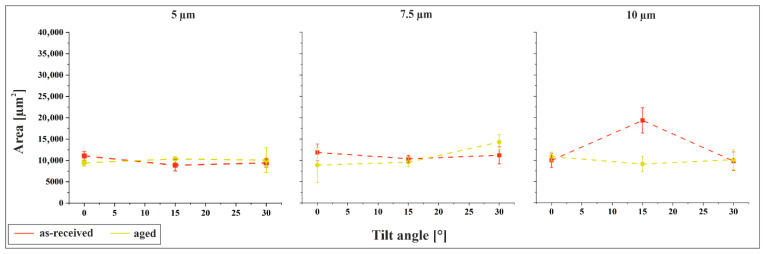
Indent size as a function of tilt angle for the as-received and the aged material, for 5 µm, 7.5 µm and 10 µm at 3 N contact force.

Figure 7 illustrates the relationship between indent area and tilt angle under a constant normal force of 3 N. Within each plot, the structural periodicity is held constant. A comparison between Figure 6 and Figure 7 reveals that the influence of tilt angle on indent size is similar to that of periodicity, with only minor variations observed as the angle increases. This suggests that tilting the sample during structuring does not significantly compromise the mechanical stability of the surface features, which is advantageous for practical applications. Notably, the primary determinant of permanent deformation is the structural periodicity rather than the flank angle, as periodicity directly governs the distribution of mechanical load.

The difference in indent size between the as-received and aged materials is generally minimal. An exception is observed for the 10 µm/15° configuration, where the as-received material exhibits indent areas nearly twice as large as those of the aged material. This behavior has been previously discussed and is attributed to the surface morphology, particularly the pronounced skewness and kurtosis that characterize the as-received sample in this configuration.

### 3.3. Contact Mechanical Considerations

**Figure 8:**
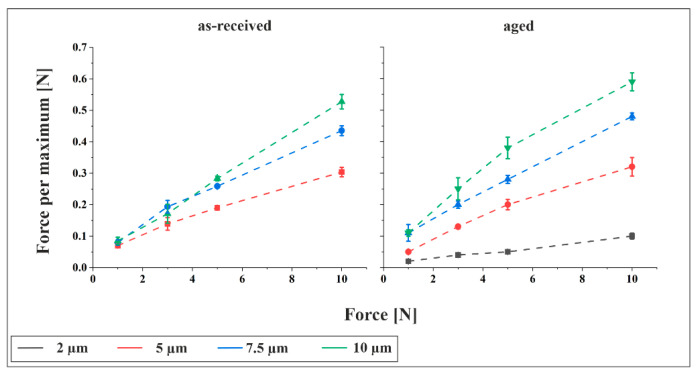
Force per maximum as a function of contact for the as-received and aged material for different periodicities at a tilt angle of 15°.

Figure 8 presents the force per structural maximum for both materials at a tilt angle of 15°. Comparable plots for tilt angles of 0° and 30° are provided in Figure S2 in the Supplementary Information, showing a consistent trend. As expected, the force per maximum increases with rising total normal force. Although higher normal forces enlarge the apparent contact area, resulting in more structural maxima and thus a broader distribution of force, this effect is outweighed by the dominant influence of the increasing normal load itself. Additionally, for similar total normal forces, structures with larger periodicities generally exhibit higher force per maximum. This is attributed to the reduced number of structural maxima in the contact zone, concentrating the force on fewer points.

**Figure 9:**
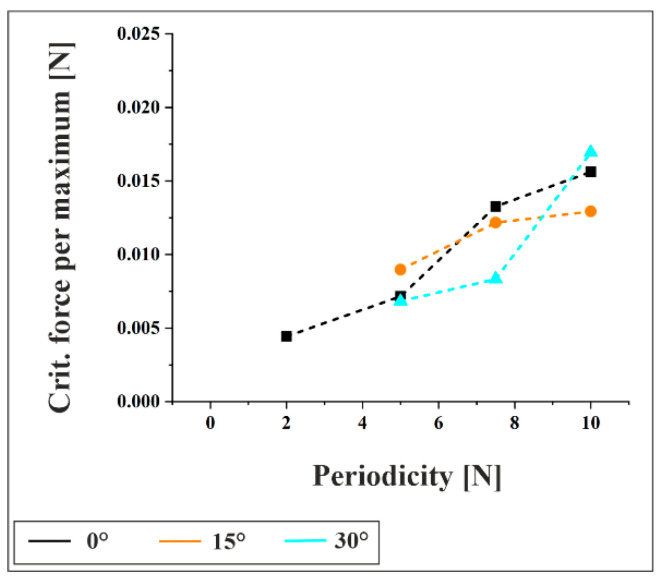
Critical load per maximum for the onset of plastic deformation, based on Equation (4), for the as-received material under a 3 N contact force.

Figure 9 displays the calculated critical load per maximum for the onset of plastic deformation, based on Equation (4), for the as-received material under a 3 N contact force. The results indicate that the critical load increases with feature size, suggesting that structures with larger periodicities are mechanically more stable and require higher loads to initiate plasticity. This trend is consistent across all tilt angles. A comparison between Figure 8 and Figure 9 reveals that the experimentally measured force per maximum significantly exceeds the calculated critical load, confirming that plastic deformation occurs under all tested configurations.

Furthermore, the critical real contact area for the onset of plasticity was calculated using Equation (5) for the as-received material at 3 N, yielding a value of 26,101 µm^2^. Although structural parameters appear in the equation, they cancel out, rendering the result independent of surface topography. Nevertheless, experimental observations clearly show that the actual contact area is strongly influenced by the surface structure. Thus, while the critical contact area for the onset of plasticity is material-dependent, the real contact area in practice is structure-dependent.

### 3.4. Simulations

**Figure 10:**
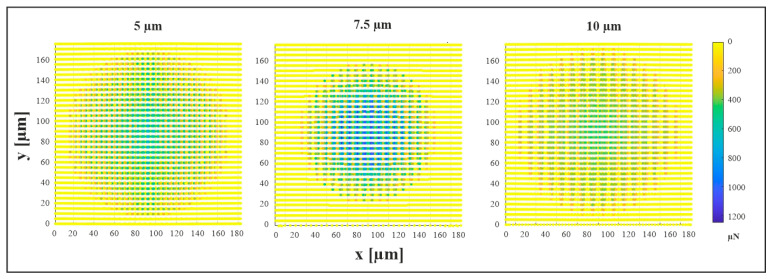
Simulated contact force distribution in µN for the as-received material in the 0° configuration at a set normal load of 3 N corresponding to the point of maximum measured force.

Figure 10 presents the simulated contact force distribution for the as-received material in the 10 µm/0° configuration under a normal load of 3 N, corresponding to the point of maximum measured force. The apparent contact area exhibits a circular geometry, whereas the actual contact area comprises multiple elliptical regions localized at topographical maxima. Subsequently, the real contact area was quantified based on these images, while the apparent contact diameter was determined post-unloading, following the procedure described earlier.

**Figure 11:**
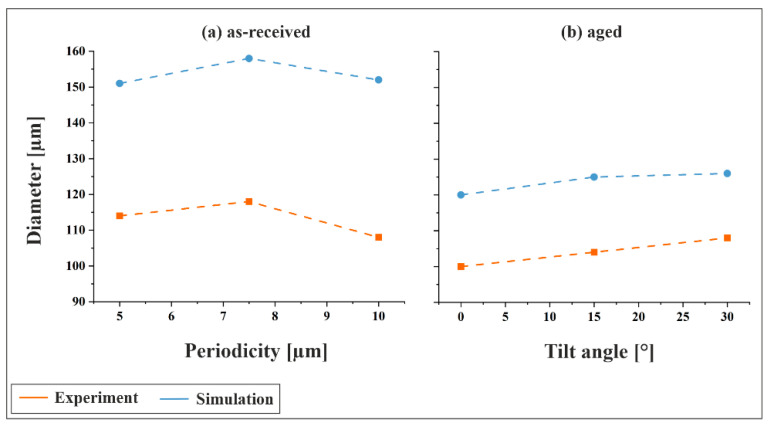
Indentation diameters obtained from experiment and simulation at 3 N as (**a**) a function of periodicity for the as-received material at a tilt angle of 0° and (**b**) as a function of tilt angle for the aged material at a periodicity of 5 µm.

Figure 11a illustrates the comparison between experimental and simulated indentation diameters for the as-received material at a tilt angle of 0°, as a function of structural periodicity. In both cases, the diameter was determined after unloading as described above. While only qualitative trends can be compared due to the empirical nature of the material model, a distinct pattern emerges: the diameter increases from 5 µm to 7.5 µm, followed by a decrease at 10 µm, approaching or slightly undercutting the value observed for 5 µm. This suggests that both small and large periodic structures exhibit greater mechanical stability, whereas intermediate-sized features are more susceptible to plastic deformation. For large periodicities, the deformation behavior is primarily governed by the high load-bearing capacity of the structures. In contrast, small periodicities benefit from a more distributed load across numerous topographical maxima, resulting in reduced stress per feature.

Figure 11b presents the comparison for the aged material using the second parameter set, focusing on the influence of tilt angle at a fixed periodicity of 5 µm. Both experimental and simulated results show a consistent trend of increasing indentation diameter with increasing tilt angle, indicating that inclined structuring leads to less mechanically stable surface topographies.

**Figure 12:**
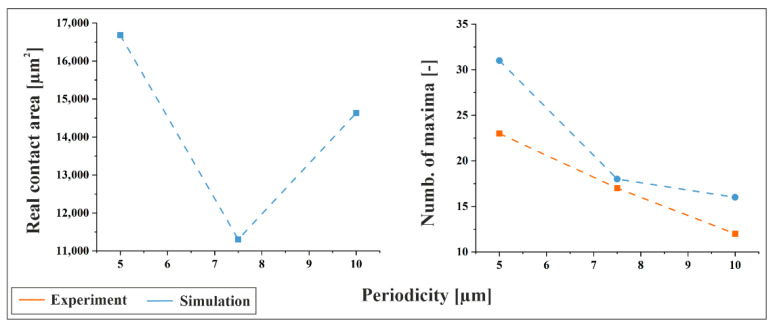
Simulated real contact area and simulated number of structural maxima within the contact zone for the as-received material at 3 N contact force. For the number of maxima, experimental values are included for comparison.

In Figure 12, the simulated real contact area and the number of structural maxima within the contact zone are shown for the as-received material. Experimental values for the number of maxima are included for comparison. The data reveal a clear trend: the number of maxima decreases with increasing periodicity, as expected. Consequently, with fewer maxima being engaged at larger feature sizes, the force per maximum increases, consistent with prior calculations.

Interestingly, the simulated real contact area (determined under load) exhibits a minimum at a periodicity of 7.5 µm, while the largest contact area is observed for the 5 µm structure. Notably, the 7.5 µm/0° configuration on as-received material also shows the largest indentation diameters (determined after unloading) in the experiments conducted at a contact force of 3 N (see Figure 6 and Figure 11a, top left, excluding the 2 µm periodicity). This indicates that the local deformation at individual topographical maxima within the contact zone is most pronounced for the 7.5 µm/0° configuration. Such behavior suggests that intermediate feature sizes may concentrate stress more effectively, leading to enhanced plastic deformation, which is beneficial for a low electrical contact resistance, since the ratio of real to nominal contact area is maximized.

### 3.5. Contact Resistance Measurements

**Figure 13:**
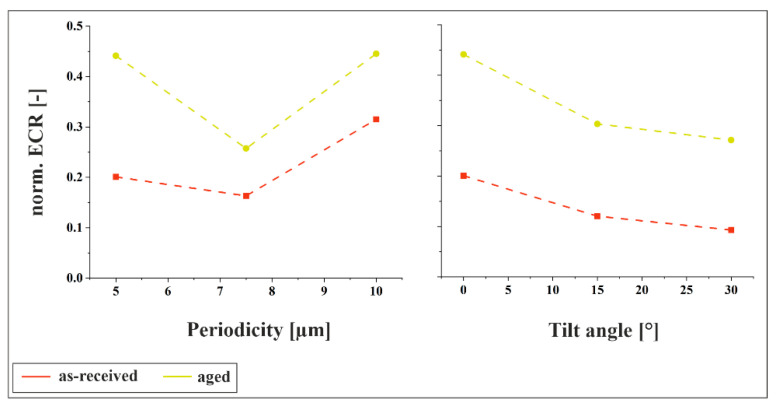
Normalized ECR values for both materials at a normal load of 3 N for 0° tilt angle and different periodicities and for 5 µm periodicity and different tilt angles.

Figure 13 presents the results of the electrical contact resistance measurements, normalized to the reference sample. For both materials, the previously observed trends are confirmed: the resistance exhibits a minimum at an intermediate periodicity of 7.5 µm and increases for both smaller and larger periodicities. Additionally, increasing the tilt angle results in a decrease in resistance, which is consistent with the larger indent sizes shown in Figure 11b.

### 3.6. Summarizing Discussion

The comparison between calculated and simulated values for the number of structural maxima and force per maximum reveals consistent trends: as periodicity increases, the number of contact points decreases, leading to higher force per maximum. This concentration of force contributes to localized plastic deformation, as confirmed by both simulations and experiments. On the other hand, larger periodicities result in structures with higher load-bearing capacity, delaying the onset of plasticity.

A notable observation is this non-monotonic behavior of indentation diameter with increasing periodicity. For the as-received material at 0° tilt, the diameter increases from 5 µm to 7.5 µm, then decreases at 10 µm. This suggests that small structures are mechanically stable due to a higher number of contact points, while large structures benefit from a broader base that distributes stress. This interpretation is supported by calculations of the critical load per maximum, which show a clear increase with periodicity, indicating that larger structures require higher loads to initiate plastic deformation. In contrast, intermediate structures (7.5 µm) lack both advantages, resulting in the largest indents and indicating reduced mechanical stability. From a design perspective, medium periodicities promote high local deformation, resulting in a low electrical contact resistance, which may be desirable for certain applications.

## 4. Conclusions

This study demonstrates that DLIP-fabricated asymmetric microstructures significantly influence the mechanical contact behavior of hot-dip tinned copper surfaces. The interplay between structural periodicity, tilt angle, and material condition governs the load-bearing capacity and plastic deformation characteristics. Among these parameters, the structural periodicity exerts a more significant impact on the permanent deformation than the flank angle, since it directly dictates the mechanical load distribution. Notably, intermediate periodicities (e.g., 7.5 µm) on as-received material at 0° tilt exhibit the highest susceptibility to plastic deformation, which is directly correlated with the lowest measured resistance values. This suggests that the pronounced plastic deformation at this periodicity enhances the real contact area, thereby reducing electrical resistance, a critical design threshold for optimizing contact performance. To further elucidate the mechanisms driving this behavior, future work should focus on localized strain and stress distribution within the microstructure, especially at topographical maxima where deformation is initiated, using experimental techniques such as digital image correlation or nanoindentation mapping and complementary simulations.

A comparison between the as-received and aged materials reveals distinct mechanical responses. The aged material, characterized by a harder Cu_6_Sn_5_ surface, generally shows reduced indent sizes and improved load-bearing capacity compared to the softer Sn surface of the as-received condition. Further, the differences between the two materials can arise from the variations in substrate composition, although the mechanical properties are comparable. However, specific configurations, such as the 2 µm/15° structure, demonstrate that aged surfaces can still exhibit significant deformation under high loads, influenced by surface morphology and roughness. This behavior is consistently observed across all tested force levels, with similar trends also evident in the untilted 2 µm structures on aged material.

Furthermore, the unexpected increase in indent size with periodicity for 15°–tilted as-received samples highlights the sensitivity of surface morphology to processing parameters and warrants further investigation. These findings underscore the importance of tailored surface structuring for optimizing contact performance in electrical applications and warrant further investigation into subsurface deformation mechanisms.

## Figures and Tables

**Table 1:** Material properties of Sn used in contact mechanical calculations.

Property	Value	Reference
Elastic modulus *E*	46.9 × $10^{9}$ Pa	[23]
Poisson ratio *ν*	0.33	[24]
Yield Strength *S_y_*	0.08 × $10^{9}$ Pa	[23]

## Data Availability

The raw data supporting the findings of this study are publicly available under https://doi.org/10.5281/zenodo.17250839 at Zenodo.

## Supplementary Materials

The following supporting information can be downloaded at: https://www.mdpi.com/article/10.3390/ma18235278/s1, Figure S1: Geometrical evaluation of the contact zones for 10 N contact force in (a) the intensity image of the 2µm/15° structure on aged material and (b) the height image of the aged reference, obtained from CLSM data. Figure S2: Force per maximum as a function of contact for the as-received and aged material for different periodicities at tilt angles of 0° and 30°.
