# Supplementary material for: Deformation Behavior of Asymmetric Direct Laser Interference Patterning Structures on Hot-Dip Tinned Copper
Source: Materials (Basel). 2025 Nov 22;18(23):5278. doi: 10.3390/ma18235278 (PMC12693050; doi:10.3390/ma18235278)
Supplement: Supplementary file 1 [file materials-18-05278-s001.zip › materials-3930920-supplementary.pdf]

Supplementary Information

Deformation Behavior of Asymmetric Direct Laser Interference Patterning Structures on Hot-Dip Tinned Copper

Silas Schütz [1,*], Sebastian Suarez [1,*], Yannik Bautz [1], Prateek Sharma [1,2], Stefan Diebels [1] and Frank Mücklich [1]

[1] Department of Materials Science and Engineering, Saarland University, 66123 Saarbrücken, Germany.

[2] Department of Mechanical and Civil Engineering, Helmut Schmidt University, 22043 Hamburg, Germany.

[*] Correspondence: silas.schuetz@uni-saarland.de (S.S.), s.suarez@mx.uni-saarland.de (S.S.)

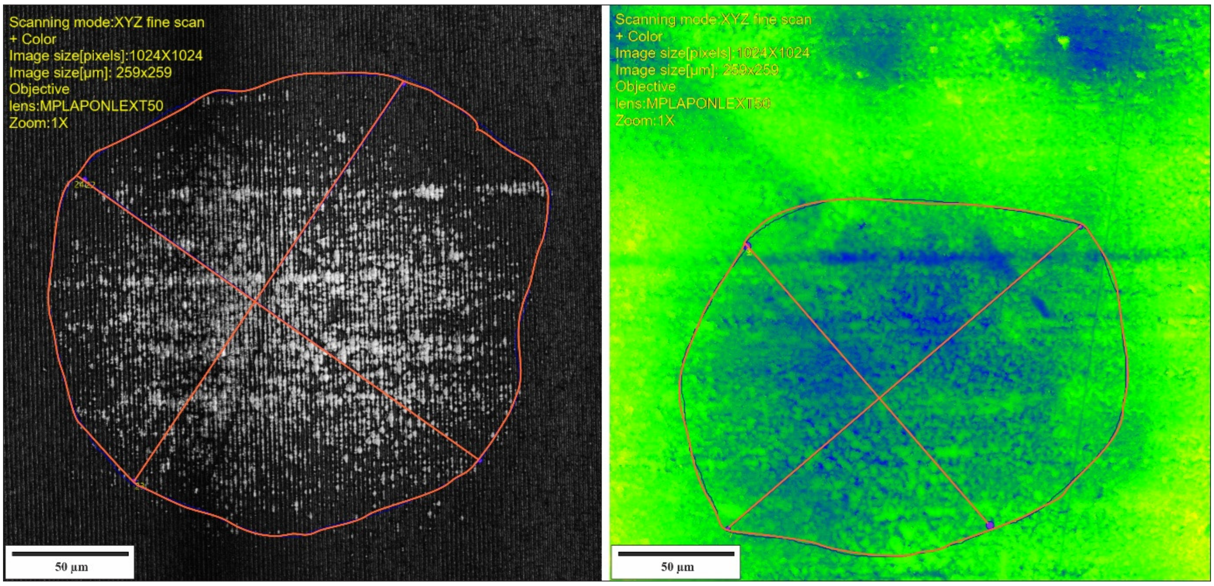

Figure. S1. Geometrical evaluation of the contact zones for 10 N contact force in **(a)** the intensity image of the 2µm/15° structure on aged material and **(b)** the height image of the aged reference, obtained from CLSM data.

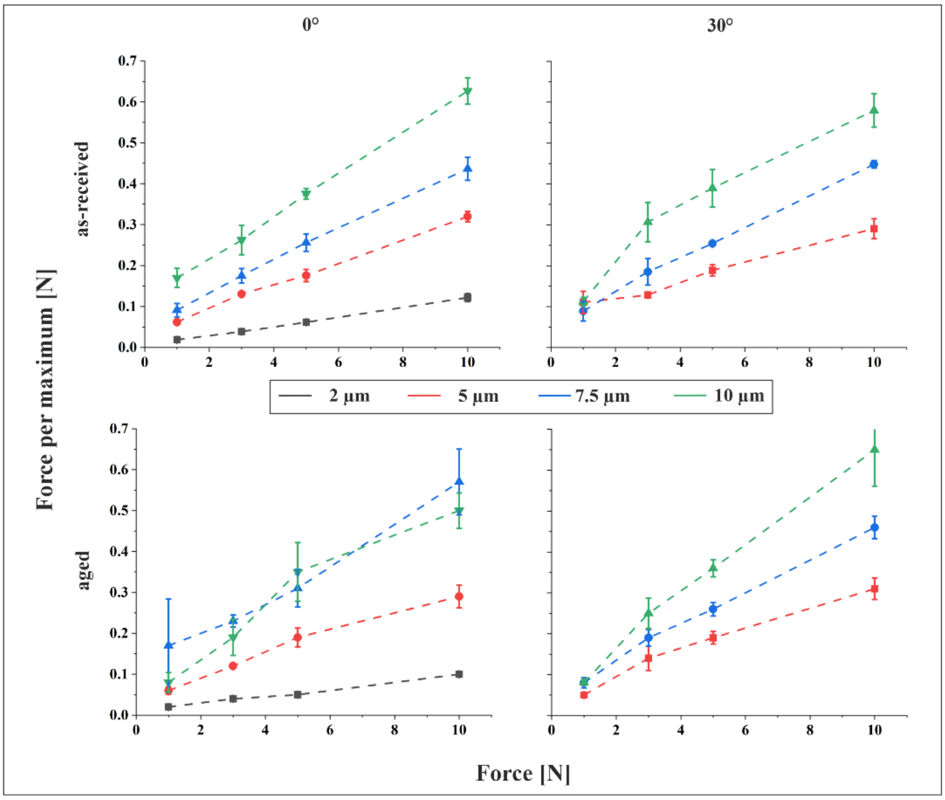

Figure. S2. Force per maximum as a function of contact for the as-received and aged material for different periodicities at tilt angles of 0° and 30°.
